# Protocol for quantifying N-Myc and global protein translation in neuroblastoma cells using click chemistry on polyvinylidene fluoride membranes

**DOI:** 10.1016/j.xpro.2024.103377

**Published:** 2024-10-11

**Authors:** Pamorn Chittavanich, Duangporn Saengwimol, Ariestya Indah Permata Sari, Atthapol Srimongkol, Rossukon Kaewkhaw

**Affiliations:** 1 Program in Translational Medicine, Faculty of Medicine Ramathibodi Hospital, Mahidol University, Bangkok 10400, Thailand; 2 Research Center, Faculty of Medicine Ramathibodi Hospital, Mahidol University, Bangkok 10400, Thailand; 3 Chakri Naruebodindra Medical Institute, Faculty of Medicine Ramathibodi Hospital, Mahidol University, Samut Prakan 10540, Thailand

**Keywords:** Cancer, Protein Biochemistry, Proteomics

## Abstract

*MYCN* amplification is a hallmark of aggressive neuroblastoma, driving N-Myc overexpression and enhancing protein synthesis, making these processes potential therapeutic targets. Here, we present a protocol for quantifying nascent N-Myc and global protein translation in neuroblastoma cells. This protocol describes the steps for labeling nascent proteins and performing an optimized click chemistry reaction directly on the membrane after blotting, enabling high-sensitivity detection and analysis. Adaptable to other proteins of interest, this approach provides valuable insights into neuroblastoma protein synthesis.

For complete details on the use and execution of this protocol, please refer to Chittavanich et al.^1^

## Highlights

• Comprehensive protocol for quantifying N-Myc and global protein translation
• Steps for the on-PVDF membrane click reaction to detect L-HPG-labeled proteins
• Guide for using immunoprecipitation to analyze N-Myc dynamics
• Detailed quantification of detected protein levels

## Abstract (editor highlights)

Publisher’s note: Undertaking any experimental protocol requires adherence to local institutional guidelines for laboratory safety and ethics.

## Graphical abstract

**Figure:**
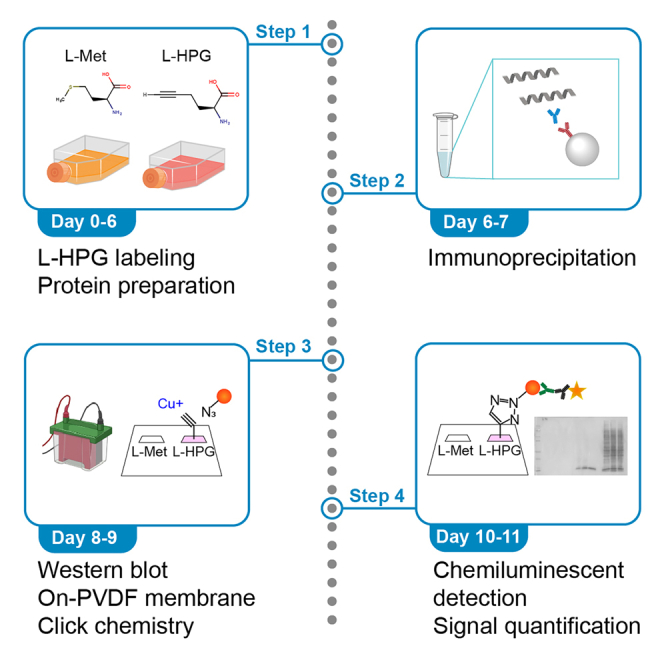

## Before you begin

This protocol details the process of quantifying newly synthesized N-Myc and global proteins in SK-N-BE (2) neuroblastoma cells, a model for aggressive neuroblastoma with *MYCN* amplification, to assess the efficacy of drugs^1^ or small molecules in inhibiting protein synthesis as a potential therapeutic strategy.^2^ We introduce an integrated immunoblotting tool to accurately identify and quantify newly synthesized proteins labeled with a clickable amino acid analog, L-homopropargylglycine (L-HPG), enabling their distinction from pre-existing proteins.

Our streamlined protocol allows for the Click reaction to occur directly on the polyvinylidene fluoride (PVDF) membrane, onto which the proteins have been transferred via Western blotting, thus simplifying the procedure compared to others.^3^ Additionally, we present an optimized Click reaction that leads to an improved signal-to-noise ratio for signal detection, resulting in a ratio greater than 50 for SK-N-BE (2) cells. Moreover, our protocol, incorporating immunoprecipitation (IP), enables the detection of specific protein translation events, such as newly synthesized N-Myc, which cannot be differentiated from pre-existing N-Myc pools using by microscopy,^3^^,^^4^ flow cytometry^3^^,^^5^ or spectroscopy^6^ for signal visualization. While demonstrated here for neuroblastoma cells, this protocol has also been successfully applied to patient-derived cancer organoids.^1^^,^^7^

We present a simple and cost-effective method for processing up to 28 samples on a single 8.5 cm × 7.0 cm membrane. The protocol comprises six key steps: (1) cell culture and nascent protein labeling, (2) protein preparation, (3) immunoprecipitation assay, (4) Western blotting, (5) Click chemistry reaction on the PVDF membrane, and (6) nascent N-Myc and global protein detection and quantification. To demonstrate the protocol’s efficacy, we utilized cycloheximide (CHX), a protein synthesis inhibitor, in a neuroblastoma cell line. As expected, we observed a reduction in the signal for both N-Myc and global proteins under CHX treatment.

### Preparation of stock solution of small molecules

**Timing: 1–2 h**

***Note:*** All reagents used with cells must be prepared under sterile conditions in a Biosafety Cabinet (BSC) level 2. Cell culture-grade dimethyl sulfoxide (DMSO) is used for small molecule preparation, and molecular biology-grade DMSO is used for Click reaction.

1. Prepare a 20 mM stock solution of L-HPG.
  a. Reconstitute L-HPG·HCl powder in ultrapure water (e.g., Milli-Q water) to 20 mM (25 mg/7.64 mL) and vortex until fully dissolved.
  b. Sterilize through a 0.22 μM filter.
  c. Aliquot and store at 4°C for short-term use (up to 3 months) and −20°C for long-term storage for up to 2 years.
2. Prepare a 20 mM stock solution of L-Methionine (L-Met).
  a. Reconstitute L-Met powder in Milli-Q water to 20 mM (25 mg/8.375 mL) and vortex until fully dissolved.
  b. Sterilize through a 0.22 μM filter.
  c. Aliquot and store at 4°C for short-term use (up to 3 months) and −20°C for long-term storage for up to 2 years.

***Note:*** The stock concentration of L-HPG and L-Met can be prepared up to 764 and 335 mM, respectively. However, the volume loss during filtering is about 0.8–1.0 mL. Therefore, we recommend preparing the concentration to produce more than 2.0 mL volume.

3. Prepare a 10 mg/mL (35.5 mM) cycloheximide (CHX) stock solution.
  a. Reconstitute CHX powder in DMSO to 10 mg/mL and vortex until dissolved.
    ***Alternatives:*** CHX can be reconstituted in ethanol at a maximum concentration of 14 mg/mL.
  b. Aliquot and store at −20°C for up to 3 years.
4. Prepare a 25 mM stock solution of MG-132.
  a. Reconstitute 10 mg of MG-132 powder in 841 μL DMSO and vortex until dissolved.
  b. Aliquot and store at −20°C for up to 3 years.
5. Prepare a 1 mM stock solution of TAMRA-azide.
  a. Reconstitute TAMRA-azide in DMSO to 1 mM (1 mg/1.583 mL). Vortex until fully dissolved.
  b. Aliquot, wrap with aluminum foil to protect from light and store at −20°C for up to 2 years.
6. Prepare a 0.02 M stock solution of phenylmethylsulfonyl fluoride (PMSF).
  a. Dissolve 3.5 mg of PMSF in 1 mL isopropanol.
  b. Aliquot, cover with parafilm, and store at −20°C for up to 1 year.

***Note:*** PMSF slowly degrades in the presence of water.

### SK-N-BE (2) cell culture media preparation

**Timing: 1–2 h**

7. Prepare 1× phosphate-buffered saline (PBS) by diluting 10× PBS in Milli-Q water and autoclaving.
8. Prepare complete Dulbecco’s Modified Eagle Medium/Nutrient Mixture F-12 (DMEM/F12) media for the cell culture of SK-N-BE (2), L-HPG labeling RPMI-1640 media for nascent protein labeling, and L-Met supplemented RPMI-1640 media for background control. Please refer to the recipes for SK-N-BE (2) cell culture media preparation.

### SK-N-BE (2) cell culture activation and maintenance

**Timing: 1–2 h**

9. Adjust the incubator’s temperature to 37°C and CO₂ to 5%, and set the water bath to 37°C.
10. Warm the culture media at 37°C for 30 min before starting cell culture.
11. Thaw cells in a cryovial at 37°C in a water bath until only a small amount of ice remains in the tube.
  a. Slowly add 1 mL of warm DMEM/F12 media into the tube.
  b. Transfer the cell suspension into a 15 mL conical tube and gently add 4 mL of DMEM/F12 media.
  c. Centrifuge the cell suspension at 300 g at 4°C for 5 min. Discard the supernatant.
  d. Resuspend the cell pellet in 1 mL DMEM/F12 media.
12. Plate them into the culture vessels containing media (e.g., 10 mL for a T75 flask).
13. Maintain cells in the incubator, with medium change every three days.

***Note:*** Before starting the experiment, maintain SK-N-BE (2) for at least one passage. When seeded at a passage ratio of 1:12 to 1:20 (or seeded at 2 × 10^6^ cells in a T75 flask), they typically reach confluence within 1–2 weeks.

### Preparation of Western blot reagents

**Timing: 3–5 h**

14. Prepare the reagents for Western blot, including 10× tris buffered saline (TBS), immunoprecipitation (IP) lysis buffer, 4× Laemmli sample buffer, 10× running buffer, 5× Tris-glycine buffer, 1× transfer buffer, 1× blocking buffer, and washing buffer (please see recipes for the preparation of Western blot reagents).
15. Prepare 10% acetic acid for HRP inactivation. Dilute 50 mL of glacial acetic acid in 450 mL of distilled water in a fume hood. Store at 25°C for up to 3 years.

**CRITICAL:** Glacial acetic acid is corrosive, flammable, and can cause severe skin burns and eye damage. Keep it from heat sources, wear appropriate personal protective equipment (PPE), and work in a fume hood.

***Alternatives:*** 25 mM glycine-HCl, pH 2.2, supplemented with 1% sodium dodecyl sulfate (SDS) can be used.

16. Prepare PVDF membranes (cut to dimensions 8.5 mm x 6.0–7.0 mm) and thick and thin filter papers.

### Preparation of reagents for casting acrylamide gel

**Timing: 1–2 h**

17. Prepare 40 mL of 6.0 M NaOH for pH adjustment.
  a. Weigh out 9.6 g of NaOH and dissolve it in distilled water.
  b. Adjust the final volume to 40 mL with distilled water in a 50 mL conical tube. Store it at 25°C for up to 3 years.

**CRITICAL:** Sodium hydroxide (NaOH) is a highly corrosive, hygroscopic pellet that can severely burn the skin and eyes. Inhalation may irritate the respiratory system and may cause pulmonary edema. Use appropriate PPE.

18. Prepare 100 mL of 3.74 M tris(hydroxymethyl)aminomethane (Tris base), pH 8.8.
  a. Weigh out 45.3 g of Tris base and dissolve it in 40 mL of distilled water.
  b. While stirring, slowly add approximately 12.3 mL of 37% HCl (12.1 M) until the pH reaches approximately 8.0.
  c. Adjust the pH to 8.8 by slowly adding 6.0 M NaOH (approximately 14 mL) while stirring.
    ***Note:*** Tris base at pH 8.8 takes a long time to dissolve. We suggest bringing the pH down to 8.0–8.4 by adding concentrated HCl solution and then adjusting to pH 8.8 using NaOH.
    **CRITICAL:** Concentrated HCl is highly corrosive and hazardous. Always wear appropriate PPE and work in a fume hood. Use appropriate transfer techniques, such as using a glass pipette. Store HCl in a cool, dry, well-ventilated area away from incompatible substances.
  d. Adjust the final volume to 100 mL and store it at 25°C for three years.
19. Prepare 40 mL of 1.25 M Tris, pH 6.8.
  a. Weigh out 6.06 g of Tris base and dissolve it in 20 mL of distilled water.
  b. Adjust the pH to 6.8 by slowly adding approximately 3.94 mL of 37% HCl.
  c. Adjust the final volume to 40 mL and store it at 25°C for three years.
20. Prepare 40 mL of 10% SDS.
  a. Weigh out 4.0 g of SDS and dissolve it in distilled water.
  b. Once completely dissolved, adjust the final volume of the solution to 20 mL with distilled water. Store it at 25°C for up to 3 years.

***Note:*** SDS solutions may become cloudy upon storage, which does not affect the solution's functionality. Warming the solution can resolve the cloudiness.

**CRITICAL:** SDS causes skin and eye irritation and may cause respiratory irritation. Wear appropriate PPE while weighing.

21. Prepare 20 mL of 10% ammonium persulfate (APS).
  a. Weigh out 2.0 g of APS and dissolve it in distilled water.
  b. Once completely dissolved, adjust the final volume of the solution to 20 mL with distilled water.
  c. Aliquot into 1 mL portions and store at −20°C for up to 1 year.

**CRITICAL:** APS powder should be kept in a desiccator. Moist air slowly induces APS decomposition, resulting in the loss of its activity. APS is a strong oxidizer. Keep it away from heat sources.

### Casting acrylamide gel for SDS-PAGE

**Timing: 2–3 h**

***Alternatives:*** 7.5% Mini-PROTEAN TGX Precast Protein Gel (BIO-RAD, #4561025 or #4561026) or any other commercial 7.5% precast polyacrylamide gel can be used.

In this section, we provide the recipe for casting a pair of gels comprising a 7.5% resolving gel and a 4.5% stacking gel. These gels effectively resolve N-Myc, global nascent protein, and β-actin. The percentage of acrylamide gel can be adjusted for proteins of different sizes.

**Figure 1:**
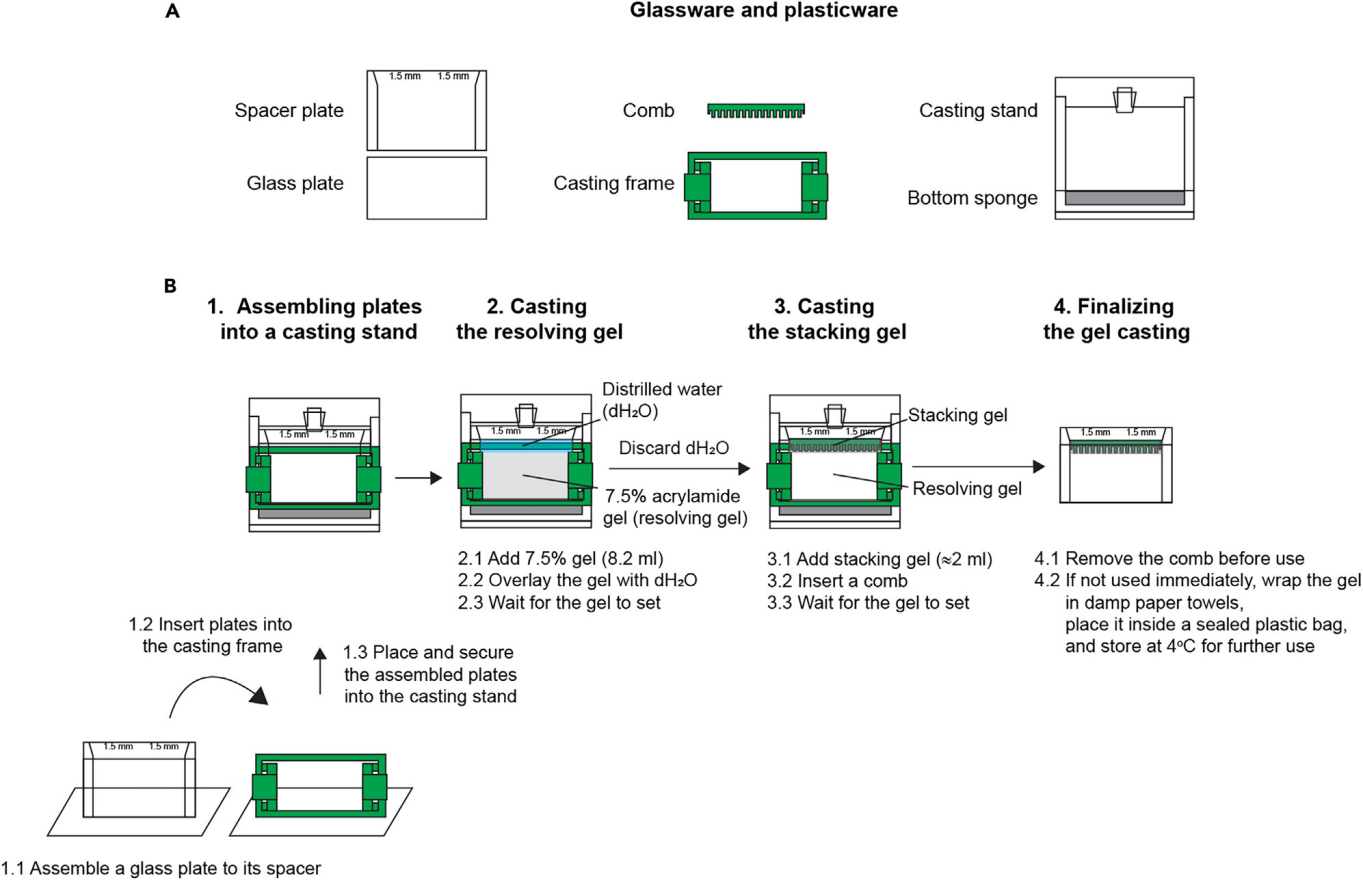
Schematic representation of SDS-PAGE gel casting (A) Glassware and plasticware for gel casting setup. (B) Step-by-step visual representation of the gel casting process.
23. Prepare the resolving gel solution according to recipes for casting acrylamide gel for SDS-PAGE, then mix well.
24. Transfer approximately 8.2 mL of resolving gel into each casting plate and gently overlay with 1 mL of distilled water (Figure 1B). Wait for about 30–60 min until the gel sets.
25. Prepare the stacking gel solution according to recipes for casting acrylamide gel for SDS-PAGE, then mix well.
26. Remove the water layer from the resolving gel, pipette the stacking solution onto the plate, insert the 1.5 mm comb (15 wells), and allow it to solidify for about 60 min (Figure 1B).

***Note:*** Wrap the gel in damp paper towels and place it inside a sealed plastic bag to prevent drying out. The gel can be stored at 4°C for up to 2 weeks.

22. Assemble two sets of glass plates into the casting stand. Ensure each set is aligned correctly and firmly seated on the bottom sponge to prevent leakage (Figures 1A and 1B).

## Key resources table

**Table:** 

REAGENT or RESOURCE	SOURCE	IDENTIFIER
**Antibodies**		

Anti-c-Myc/N-Myc (D3N8F) rabbit mAb (IP 1:400, WB 1:1,000)	Cell Signaling Technology	Cat# 13987S, RRID: AB_2631168
Rabbit IgG isotype control (IP 0.5 μg/mL or 1:10,000)	Invitrogen	Cat# 02-6102, RRID: AB_2532938
Anti-TAMRA monoclonal antibody (5G5) (WB 1:1,000)	Invitrogen	Cat# MA1-041, RRID: AB_2536728
Anti-mouse IgG, HRP-linked antibody (WB 1:10,000)	Cell Signaling Technology	Cat# 7076, RRID: AB_330924
Anti-rabbit IgG, HRP-linked antibody (WB 1:3,000)	Cell Signaling Technology	Cat# 7074, RRID: AB_2099233
β-actin (8H10D10) mouse mAb (HRP conjugate) (WB 1:10,000)	Cell Signaling Technology	Cat# 12262, RRID: AB_2566811

**Chemicals, peptides, and recombinant proteins**		

L-homopropargylglycine hydrochloride (L-HPG·HCl) **Note:** Click Chemistry Tools is now part of Vector Laboratories	Click Chemistry Tools	Cat# 1067-25
L-methionine (L-Met)	Sigma-Aldrich	Cat #M5308
Cycloheximide (CHX)	Tocris Bioscience	Cat# 0970100
Dimethyl sulfoxide (DMSO), plant cell culture grade	Sigma	Cat# D4540
Dimethyl sulfoxide (DMSO), laboratory-grade	Merck	Cat# 1167431000
MG-132	Merck	Cat# 474790
Azide-fluor 545 (TAMRA azide)	Sigma-Aldrich	Cat# 760757
Phenylmethylsulfonyl fluoride (PMSF)	Sigma-Aldrich	Cat# P7626
Isopropanol	Merck	Cat# 109634
10× phosphate-buffered saline (10× PBS)	HyClone	Cat# 3025802
Dulbecco’s modified Eagle’s medium (DMEM)/F12 1:1 with L-glutamine and HEPES	HyClone	Cat# SH3002302
Fetal bovine serum (FBS)-ES cell qualified	Merck	Cat# ES-009-B
Penicillin-streptomycin 100× solution	Invitrogen	Cat# 15140-122
RPMI-1640 without methionine	Invitrogen	Cat# A1451701
TrypLE express enzyme	Invitrogen	Cat# 12604021
Halt protease inhibitor cocktail (100×)	Invitrogen	Cat# 78430
Tris(hydroxymethyl)aminomethane (Tris-base)	Vivantis	Cat# PR0612
Sodium chloride (NaCl)	Merck	Cat# 106404
37% hydrochloric acid (HCl)	Sigma-Aldrich	Cat# 258148
NP-40	Merck	Cat# 492016
OmniPur ethylenediaminetetraacetic acid (EDTA), disodium salt, dihydrate	Merck	Cat# 4010
UltraPure 0.5 M EDTA, pH 8.0	Invitrogen	Cat# 15575020
Glycerol	Merck	Cat# 4750
Bromophenol blue	Sigma-Aldrich	Cat# 114391
Sodium dodecyl sulfate (SDS)	Merck	Cat# 7910
Glycine	Bio-Rad	Cat# 161-0718
Methanol	Merck	Cat# 106009
Bio-Rad blocking grade non-fat dry milk	Bio-Rad	Cat# 1706404
Tween 20	Merck	Cat# 9480
Sodium hydroxide (NaOH)	Merck	Cat# 106498
Ammonium persulfate (APS)	Merck	Cat# 2300
Glacial acetic acid	Merck	Cat# 100063
30% acrylamide gel	Bio-Rad	Cat# 161-0156
Tetramethylethylenediamine (TEMED)	Merck	Cat# 8920
Protein A/G plus agarose	Santa Cruz Biotechnology	Cat# sc-2003
Protein ladder (Bio-Rad Precision Plus protein dual color standard)	Bio-Rad	Cat# 161-0374
Aminoguanidine hydrochloride (AGD·HCl)	Sigma-Aldrich	Cat# 396494
Sodium ascorbate	Sigma-Aldrich	Cat# A4034
Trypan blue solution, 0.4%	Gibco	Cat# 15250061
β-mercaptoethanol	Sigma-Aldrich	Cat# M6250
Immobilon Forte western HRP substrate	Millipore	Cat# WBLUF0100

**Critical commercial assays**		

Pierce BCA protein assay kit	Thermo Fisher Scientific	Cat# 23227

**Experimental models: Cell lines**		

SK-N-BE (2) cells	ATCC	CRL-2271, RRID: CVCL_0528

**Software and algorithms**		

Image Lab (v.6.1)	Bio-Rad	https://www.bio-rad.com/en-th/product/image-lab-software Cat# SOFT-LIT-170-9690-ILSPC-V-6-1 for Window
GraphPad Prism (v. 9.5)	GraphPad	https://www.graphpad.com

**Other**		

EasYFlask cell culture flasks (T75)	Thermo Scientific Nunc	Cat# 156499
6-well clear TC plate (6-well plate)	Corning	Cat# 3516-50
Immun-Blot PVDF membrane, roll, 26 cm × 3.3 m	Bio-Rad	Cat# 1620177
Thick Blot filter paper, precut, 18 × 34 cm	Bio-Rad	Cat# 1650921
Whatman filter paper no.1 (thin filter)	Whatman	Cat# 1001-110
Mini-PROTEAN spacer plates with 1.5 mm integrated spacers	Bio-Rad	Cat# 1653312
Glass plates	Bio-Rad	Cat# 1702982
Mini-PROTEAN tetra cell casting stand with clamp kit for single core system	Bio-Rad	Cat# 1658052
Mini-PROTEAN combs, 15-well, 1.5 mm, 40 μL	Bio-Rad	Cat# 1653366
96-well clear TC plate (96-well plate)	Corning	Cat# 3599
Microplate reader	Tecan Trading	Infinite M200 Pro
Heating block	Major Science	Cat# 1MJC-EL-02-220
PowerPac basic power supply **Note:** PowerPac HC power supply Cat# 1645052 can be used	Bio-Rad	Cat# 1645050
Mini Trans-Blot electrophoretic transfer cell	Bio-Rad	Cat# 1703930
Rocker-Shaker	Hercuvan Lab Systems	TT-10-RS-1/TT-10-RS-2 2D
Vortex-Genie 2	Scientific Industries	SKU: SI-0236
ChemiDoc MP imaging system with Image Lab software	Bio-Rad	Cat# 12003154
Neubauer improved, bright-line, double ruling, with 2 coverglass (hemocytometer)	BOECO	Cat# BOE14
Acrodisc syringe filters with Supor membrane, sterile - 0.22 μm, 32 mm	Pall	Part No. 4652
RS-60 tube rotator	Biosan	BS-010118

## Materials and equipment

### Recipes for SK-N-BE (2) cell culture media preparation

**Table:** DMEM/F12 media

Reagent	Final concentration	Amount
100× Penicillin-Streptomycin	1×	0.5 mL
FBS	10%	5 mL
DMEM/F12	1×	44.5 mL
**Total**	**1**×	**50 mL**

Store at 4°C for up to one month.

**Table:** RPMI-1640 media

Reagent	Final concentration	Amount
100× Penicillin-Streptomycin	1×	0.5 mL
FBS	15%	7.5 mL
20 mM L-methionine	An additional 100 μM is added to the medium, resulting in a total concentration of 200 μM.	0.25 mL
RPMI-1640	1×	41.75 mL
**Total**	**1**×	**50 mL**

Store at 4°C for up to one month.

***Note:*** The concentration of L-Met is the same as that of the protein labeling agent L-HPG.

***Alternatives:*** RPMI-1640 without methionine supplemented with 200 μM L-Met can be used.

**Table:** L-HPG labeling RPMI-1640 media

Reagent	Final concentration	Amount
100× Penicillin-Streptomycin	1×	0.5 mL
FBS	15%	7.5 mL
20 mM L-HPG	200 μM	0.5 mL
RPMI-1640 without methionine	1×	41.5 mL
**Total**	**1**×	**50 mL**

Store at 4°C for up to one month.

***Note:*** Refer to Figure S1 for the effect of L-HPG concentration on the labeling of nascent proteins.

### Recipes for the preparation of Western blot reagents

**Table:** 10× Tris-buffered saline, pH 7.4 (10× TBS)

Reagent	Final concentration	Amount
Tris-base	0.25 M	30.4 g
NaCl	1.5 M	87.8 g
37% HCl (12 M)	To pH = 7.4	≈17.4 mL
Distilled water	N/A	To 1L
**Total**	**10**×	**1 L**

Autoclave and store at 25°C for up to 3 years.

***Note:*** The amount of HCl can be estimated using the Henderson-Hasselbalch Calculator for Tris Buffer's webpage.

***Alternatives:*** 10× TBS (Fisher Scientific, Cat. No. BP24711) or any commercial 10× TBS.

**CRITICAL:** Hydrochloric acid (HCl) is a highly corrosive liquid and vapor, causing severe skin burns and eye damage. It may also cause respiratory irritation. Always wear appropriate PPE and work in a fume hood.

**Table:** Immunoprecipitation (IP) lysis buffer

Reagent	Final concentration	Amount
10X TBS	1×	4 mL
NP-40	1% v/v	0.4 mL
0.5M EDTA	1 mM	80 μL
Glycerol	5% v/v	2 mL
Distilled water	N/A	≈33.5 mL
**Total**	**1**×	**40 mL**

Autoclave and store at 4°C for up to 2 years.

Add 10 μL of 100× Halt Protease inhibitor cocktail to 0.99 mL of autoclaved IP lysis buffer.

***Alternatives:*** Pierce IP Lysis buffer (Thermo Scientific, Cat No 87787). Use Halt Protease and phosphatase Inhibitor Cocktail (100×) (Thermo Scientific, Cat No 78442) if phosphorylation species of N-Myc are studied.

**Table:** 4× Laemmli sample buffer

Reagent	Final concentration	Amount
Bromophenol Blue	0.022% w/v	8.0 mg
SDS	4.4% w/v	1.6 g
Tris-base	278 mM	1.2 g
37% HCl	To pH 6.8	Adjust pH = 6.8
Glycerol	44.4% v/v	16 mL
Distilled water	N/A	Up to 36 mL
**Total**	**4.4**×	**36 mL**

Store at 25°C for up to 3 years.

***Alternatives:*** 4× Laemmli sample buffer (BIO-RAD, Cat. No. #161–0747).

To prepare 1 mL of 4× Laemmli sample buffer, add 100 μL of β-mercaptoethanol to 900 μL of this 4.4× sample buffer. Seal with parafilm and store at 4°C for up to 1 year.

**Table:** 10× running buffer

Reagent	Final concentration	Amount
EDTA	0.75% w/v	3.75 g
SDS	2% w/v	10 g
Tris-base	250 mM	15 g
Glycine	1,920 mM	72 g
Distilled water	N/A	Up to 500 mL
**Total**	**10**×	**500 mL**

Store at 4°C in the dark for up to 2 years.

***Alternatives:*** 10× Tris/Glycine/SDS buffer (Bio-Rad, Cat. No. #1610772).

To prepare 1× running buffer, dilute 100 mL of 10× running buffer with 900 mL of distilled water. Store at 25°C or 4°C in the dark for up to 1 year.

***Note:*** Using pre-chilled buffers can help maintain the integrity of samples and achieve sharper bands during electrophoresis.

**Table:** 5× Tris-glycine buffer

Reagent	Final concentration	Amount
SDS	0.05% w/v	0.5 g
Tris-base	250 mM	30 g
Glycine	1,920 mM	144 g
Distilled water	N/A	Up to 1,000 mL
**Total**	**5**×	**1,000 mL**

Store at 25°C in the dark for up to 2 years.

***Alternatives:*** Trans-blot Turbo 5× Transfer buffer (Cat #10026938).

**Table:** 1× transfer buffer

Reagent	Final concentration	Amount
5× tris-glycine buffer	1×	200 mL
Methanol	20%	200 mL
Distilled water	N/A	Up to 1,000 mL
**Total**	**1**×	**1,000 mL**

Store at 4°C in the dark for up to 1 year.

**CRITICAL:** Methanol is a highly flammable liquid and vapor. It is toxic if swallowed, comes into contact with the skin, or is inhaled. Keep it away from heat sources and wear appropriate PPE.

**Table:** Western blot-blocking buffer

Reagent	Final concentration	Amount
Non-fat dry milk	5% w/v	25 g
10× TBS	1×	50 mL
Tween-20	0.2% v/v	1 mL
Distilled water	N/A	Up to 500 mL
**Total**	**1**×	**500 mL**

Store at 4°C for up to 3 months.

**Table:** Washing buffer

Reagent	Final concentration	Amount
10× TBS	1×	50 mL
Tween-20	0.2% v/v	1 mL
Distilled water	N/A	449 mL
**Total**	**1**×	**500 mL**

Store at 4°C for up to 3 months.

### Recipes for casting acrylamide gel for SDS-PAGE

**Table:** Resolving gel

Reagent	Final concentration	Amount
30% acrylamide gel	7.5%	4.4 mL
3.74 M Tris (pH 8.8)	0.935 M	4.4 mL
Distilled water	N/A	8.3 mL
10% SDS	0.2% w/v	352 μL
10% APS	0.067% w/v	118 μL
TEMED	0.1% v/v	17.6 μL
**Total**	**1**×	**17.6 mL**

**CRITICAL:** APS and TEMED must be added last.

**Table:** Stacking gel

Reagent	Final concentration	Amount
30% acrylamide gel	5%	0.76 mL
1.25 M Tris (pH 6.8)	0.0625 M	0.5 mL
Distilled water	N/A	3.61 mL
10% SDS	0.2% w/v	100 μL
10% APS	0.067% w/v	50 μL
TEMED	0.1% v/v	5 μL
**Total**	**1**×	**5 mL**

**CRITICAL:** APS and TEMED must be added last.

## Step-by-step method details

### Cell culture and nascent protein labeling – Day 0–5

**Timing: 2 h for cell seeding, 1–4 days for cell culture, and 2 h for L-HPG labeling**

The density of cells in a culture can significantly impact the protein translation rate. To optimize this process, we recommend plating the cells at least one day before the L-HPG labeling treatment, ensuring the culture reaches 60–80% confluence by the time of labeling. The plating density and media maintenance should be adjusted based on the specific cell type.

In this protocol, we demonstrate triplicates of cultures with protein inhibitor (CHX) or DMSO (vehicle) treatments, each subjected to L-HPG labeling, alongside control cultures without L-HPG labeling [(−) L-HPG]. Additionally, we used an IgG isotype control as a negative control for the N-Myc-IP assay.

***Note:*** In organoid culture systems that require Matrigel embedding, it is essential to note that Matrigel can lead to a low background signal in the (−) L-HPG condition.

1. Plate SK-N-BE (2) cells at optimal density.
  a. Ensure cell confluency is around 70–80% at the time of passaging. Wash the cells once with 4 mL of 1× PBS.
  b. Dissociate the cell culture using 4 mL of 1× TrypLE Express Enzyme for a T75 flask. Incubate for 5–10 min. Tap the flask to detach the cells and transfer the cell suspension into a 15 mL conical tube.
  c. Add an equal volume of 1× PBS, mix, and centrifuge for 5 min at 300g. Discard the supernatant and resuspend the cell pellet in 1 mL of DMEM/F12.
  d. Dilute the cell suspension 5–10 times in a 1.5 mL tube. Mix 10 μL of the diluted cell suspension with 10 μL of 0.4% trypan blue solution. Count viable cells (excluding those stained by trypan blue) using a hemocytometer.
  e. For the quantification of N-Myc translation, we recommend a plating density of 2.0 × 10^6^ cells in T75 flasks with 10 mL of media, cultured for 4 days. This setup is intended for use in each of the following four experimental groups: CHX (35.5 μM), vehicle (0.1% DMSO), (−) L-HPG and IgG isotype control.
  f. For the quantification of global protein translation, we recommend a plating density of 0.3 × 10^6^ cells in a 6-well plate with 2 mL of media, cultured for 4 days. This setup is intended for use in each of the following three experimental groups: CHX (35.5 μM), vehicle (0.1% DMSO), and (−) L-HPG.

**Pause point:** At the mentioned density, the SK-N-BE (2) cell line requires four days to reach the desired number for the experiment.

2. On day 5, prepare a small molecule in the L-HPG labeling media.
  a. For CHX treatment, add CHX and MG-132 to L-HPG labeling RPMI-1640 media (i.e., 10 μL each small molecule per 10 mL media).
  b. For vehicle and IgG isotype control treatments, add DMSO and MG-132 to L-HPG labeling RPMI-1640 media (i.e., 10 μL each small molecule per 10 mL media).
  c. For (−) L-HPG treatment, add DMSO and MG-132 to RPMI-1640 media (i.e., 10 μL each small molecule per 10 mL media).

***Note:*** MG-132, a proteasomal inhibitor, improves the stability of N-Myc, which has a short half-life, thereby enhancing signal intensity. Use this proteasomal inhibitor specifically for the quantification of N-Myc translation. It is not required for the quantification of global protein translation.

3. Treat cells by adding the prepared media from step 2 to each experimental group.
  a. Wash the cultures briefly twice with 1× PBS.
  b. Treat the cells with the prepared media from step 2 (i.e., 10 mL for a T75 flask and 2 mL for a well of a 6-well plate) and incubate treated cells for 2 h. Troubleshooting problem 1.

***Note:*** The optimal conditions for incubation duration and concentration of L-HPG for SK-N-BE (2) cells range from 30 to 120 min and 50–200 μM, respectively (Figures S1 and S2). More extended incubation periods and higher L-HPG concentrations lead to a higher nascent protein signal.

### Protein preparation – Day 5

**Timing: 3 h**

This section outlines the procedure for extracting proteins and determining their concentration using the BCA assay.

4. Extract the proteins from the treated cells.
  a. Prepare IP lysis buffer containing 1% proteinase inhibitor cocktail (100× stock).
    ***Note:*** RIPA buffer can be an alternative to IP lysis buffer for extracting proteins in the global protein translation assay.
  b. Wash the cell cultures twice with cold 1× PBS.
  c. Lyse cells by adding prepared IP lysis buffer (300–400 μL for a T75 flask or 100 μL for a well of a 6-well plate). Incubate the lysed cells on ice for 30 min, pipetting up and down every 10 min.
  d. Transfer the lysate into a 1.5 mL tube. Centrifuge the tube at 4°C for 15 min.
  e. Transfer the supernatant to a new 1.5 mL tube.
    **Pause point:** Protein lysates can be stored at −80°C until used.
5. Thaw the samples if stored at −80°C.
6. Prepare lysate samples by diluting 10 μL of lysate with 10 μL of IP lysis buffer for a 1:1 ratio.
7. Prepare eight protein standards.
  a. Dilute 2 μg/μL BSA with distilled water to the following concentrations: 0, 0.2, 0.4, 0.8, 1.2, 1.6, 1.8, and 2.0 μg/μL.
  b. Use IP lysis buffer for samples and 0 μg/μL of BSA for standards as a blank.
8. Add 10 μL of blank, protein standards, and diluted samples to wells in a 96-well plate.
9. Prepare Pierce BCA reagent with an A: B ratio of 50:1. Calculate the total volume of the mixture needed for 200 μL in each well.
10. Add 200 μL of mixed BCA reagents to each well. Incubate at 37°C for 30 min.
11. Read the absorbance at a wavelength of 562 nm using a plate reader. Subtract the absorbance readings of the blank solution from the sample or standard reading.
12. Construct a protein standard curve with absorbance readings on the X-axis and protein concentrations on the Y-axis. Fit a linear regression to the standard curve, setting the intercept to 0, to determine the concentrations of the samples. Troubleshooting problem 2.

***Alternatives:*** Other protein determination methods, like the Bradford assay, can be used as an alternative.

13. Aliquot 500 μg protein for quantification of N-Myc translation. Store at −20°C until use.

***Note:*** The recommended input protein concentration is greater than 1 μg/μL

14. Aliquot 10–20 μg protein for quantification of global protein translation. Store at −20°C until use.

**Pause point:** The experiment can be paused here.

### Immunoprecipitation assay for N-Myc isolation – Day 6–7

**Timing: 2–4 h**

This section describes immunoprecipitation and sample elution for detecting newly synthesized N-Myc. If the analysis is focused solely on global protein translation, proceed to Western blotting: SDS-PAGE for protein separation and transfer on PVDF.

15. Thaw samples and chill them on ice.
16. Freshly prepare a 0.02 μM solution of PMSF in PBS, using 1 mL per sample.
17. Dilute protein lysate samples to a final concentration of 1.5 μg/μL using IP lysis buffer. Mix 333 μL (containing 500 μg of protein) of protein lysate with 644.5 μL of IP lysis buffer.
18. Add 2.5 μL of anti-N-Myc rabbit antibody (final concentration 1:400) or the same concentration of rabbit IgG isotype control antibody per sample and rotate the mixtures for 1 h at 4°C.

**CRITICAL:** Avoid using antibodies that recognize the high-methionine-rich regions due to the reduced binding affinity of antibodies towards L-HPG-incorporated proteins.

19. Add 20 μL of protein A/G plus agarose per sample. The final volume of the sample will become 1 mL. Rotate the samples at 4°C overnight (14–16 h) on a vertical rotator.
20. Freshly prepare 5 mL of IP washing buffer per sample according to Table 1.

**Table 1:** IP washing buffer

Reagent	Final concentration	Amount
NP-40	0.1%	25 μL
0.02 mM PMSF	0.02 μM	25 μL
10× PBS	1×	25 mL
Distilled water	N/A	Up to 25 mL
**Total**	**1**×	**25 mL**
21. Centrifuge the samples at 1,000 g for 5 min at 4°C. Discard the supernatant.
22. Wash the samples with IP washing buffer four times, using the centrifugation speed specified in step 21.
23. Resuspend the sample in 50 μL of 1× Laemmli sample buffer.
24. Boil samples at 95°C for 10 min, then centrifuge at 500 g for 5 min.
25. Transfer the supernatant to a new 1.5 mL tube. Store the samples at −20°C until use.

**Pause point:** The experiment can be paused here.

### Western blotting: SDS-PAGE protein separation and transfer on PVDF membrane – Day 8

**Timing: 4–6 h**

26. Thaw the stored samples and chill them on ice.
27. Mix the sample with 4× Laemmli sample buffer at a 3-part- to 1-part buffer ratio for global protein translation samples and boil at 95°C for 10 min.
28. Perform the Western blotting.
  a. Load the samples onto a 7.5% gel and 1.5 μL of the protein ladder (Bio-Rad Precision Plus Protein Dual Color Standard).
    i. For N-Myc translation quantification, load 20 μL per sample.
    ii. For global translation quantification (Separate gel): Load equal amounts of total protein (10–20 μg) for each sample.
      ***Note:*** We recommend leaving one well space between (−) L-HPG and the samples to minimize spillage between lanes.
  b. Start protein separation by running the gel at 50 V for 5 min. Then, increase the voltage to 100 V and continue the separation for 60–120 min in an ice box or until the marker proteins have fully separated.
    ***Optional:*** Label the PVDF membrane with a pencil to identify the loading sample set and assay types.
  c. Activate the PVDF membrane by immersing it in methanol for 5 s, followed by a 2-min incubation in distilled water, and then transfer it into the transfer buffer.
  d. Assemble the blotting cassettes in the following order: (Bottom) white side of the cassette, sponge, thick filter paper, thin filter paper, PVDF membrane, gel, thin filter paper, thick filter paper, (Top) black side of the cassette. Handle carefully to prevent bubbles between the PVDF membrane and the gel.
  e. Insert the cassettes into the electrode assembly (back facing back) and place them in the tank containing the cold transfer buffer. Add an ice block to the tank to maintain a stable temperature and prevent overheating during protein transfer.
  f. Transfer the proteins using a constant current of 400 mA for 100 min.
    ***Note:*** To verify the completeness of the transfer, examine the remaining protein ladder on the gel or stain the gel with Coomassie Brilliant Blue and inspect the bands.
  g. Submerge the membrane in autoclaved 1× PBS. The membrane can be stored at 4°C for up to one week.
    **Pause point:** The experiment can be paused here.
    **CRITICAL:** Before performing the Click reaction, avoid using TBS, as any residual solution on the membrane can potentially reduce the efficiency of the Click reaction.

### Click chemistry reaction on PVDF membrane – Day 9

**Timing: 4–6 h**

The following steps outline the procedure for performing copper(I)-catalyzed Click chemistry to conjugate TAMRA-linked azide to L-HPG incorporated in nascent proteins. The protocol includes subsequent washing, blocking, and detection using HRP-conjugated anti-TAMRA antibodies. This protocol is designed for use with pairs of 8.5 mm × 7.0 mm PVDF membranes.

29. Briefly wash the membrane twice in distilled water.
30. Prepare a 1:1 solution of DMSO and autoclaved distilled water and incubate the membrane in this solution for 5 min. The membrane becomes transparent after this DMSO treatment (Figure 2).

**Figure 2:**
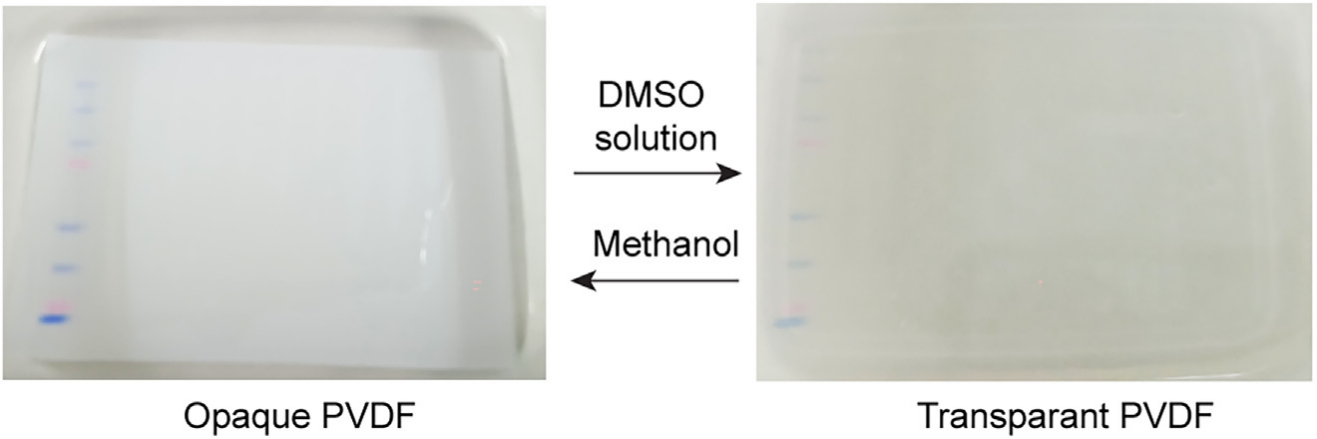
Alteration in optical properties of PVDF membrane When exposed to different solvents, a PVDF membrane undergoes a reversible change in optical properties. Immersion in a 1:1 DMSO: water solution induces a transition from opaque to transparent, while subsequent exposure to methanol reverses this effect, restoring the membrane’s original opacity.
31. Thaw the TAMRA-linked azide solution by gently agitating it at 25°C and protecting it from light.
32. Freshly prepare 100 mM aminoguanidine·HCl (AGD·HCl) (11.055 mg/mL) and 100 mM sodium ascorbate (19.811 mg/mL) in autoclaved distilled water.

**CRITICAL:** Sodium ascorbate can be oxidized by atmospheric oxygen. Fresh sodium ascorbate powder is usually white or slightly yellow. A darker color might suggest oxidation. Proper storage practices (cool, dry, dark place with a tightly sealed container) can help maintain its efficiency for longer.

***Alternatives:*** Aminoguanidine (AGD) and ascorbic acid can be used instead of AGD·HCl and sodium ascorbate.

33. Freshly prepare the Click solution according to Table 2, combining reagents in the specified order within a single tube. Prepare 6 mL of Click solution for an 8.5 cm × 7.0 mm PVDF membrane. Thoroughly mix the solution before adding sodium ascorbate, followed by vertexing. Ensure membranes and a shaker are ready for the subsequent step.

**Table 2:** Click reaction solution

Reagent	Final concentration	Amount
250 mM CuSO_4_	20 mM	960 μL
1 mM TAMRA-linked azide	10 μM	120 μL
Distilled water	N/A	2.64 mL
DMSO	49% (6.9 M)	5.88 mL
100 mM AGD	10 mM	1.2 mL
100 mM sodium ascorbate	10 mM	1.2 mL
**Total**	**1**×	**12 mL**

**Figure 3:**
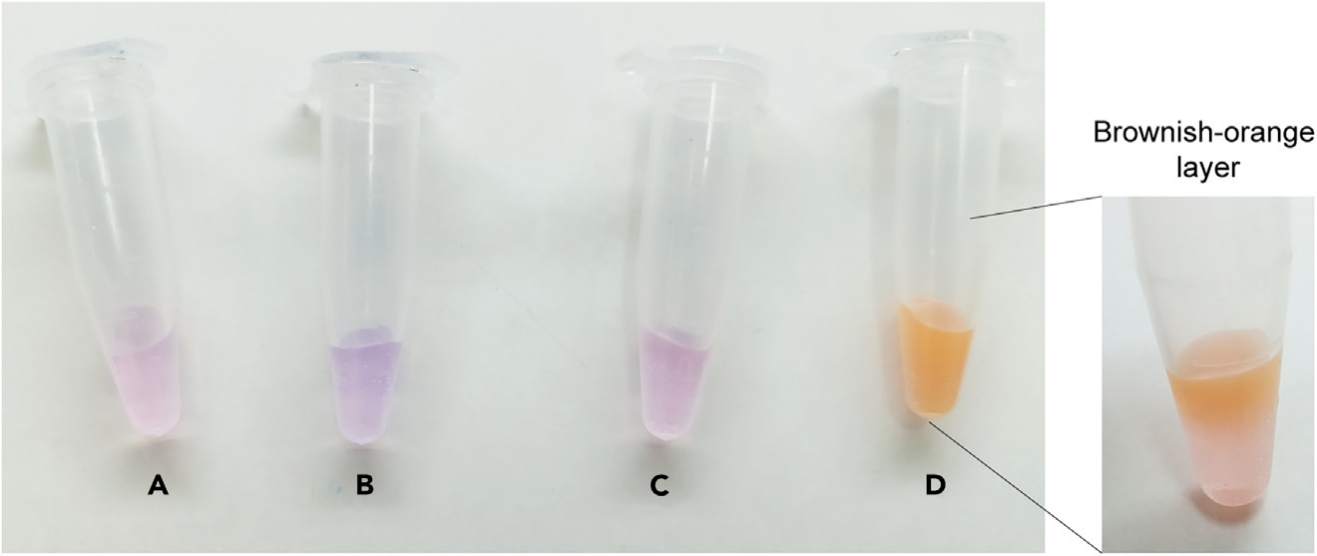
Color changes at each stage in the Click reaction solution See also Table S1. (A) The CuSO_4_ and TAMRA-linked azide mixture yields a light pink solution. (B) The addition of AGD to the solution (CuSO_4_ + TAMRA-linked azide + AGD) results in a bluish color. (C) Mixing with ascorbate causes the solution (CuSO_4_ + TAMRA-linked azide + AGD + sodium ascorbate) to return to pink. (D) In the absence of AGD, CuSO_4_, TAMRA-linked azide, and sodium ascorbate solution form a brownish-orange layer.

***Note:*** Prepare the Click reaction solution by adding CuSO_4_ first, followed by the remaining reagents in Table 2. Add sodium ascorbate last to the same tube. The 250 mM CuSO_4_ (BCA reagent B, 4% CuSO_4_) from the Pierce BCA Protein Assay Kit can be used. This protocol has been optimized and modified based on the study by Ohata.^8^ Please refer to Figures 3A–3C for the color development after adding each reagent. If AGD is not added, the reaction produces a brownish-orange color layer (Figure 3D). For details on optimization and the role of each reagent, please see Figures S3, S4, and Table S1 in the supplementary materials.

34. Incubate the membrane in 6 mL of the Click reaction solution with agitation for 1 h at 25°C, protecting it from light.

***Note:*** 6 mL of the Click reaction solution is sufficient for an 8.5 mm × 7.0 mm PVDF membrane.

**CRITICAL:** The Click reaction solution must be used within 10 min after adding sodium ascorbate.

35. After 1 h, discard the Click reaction solution. Briefly wash the membrane with methanol until it becomes opaque (original PVDF state) (Figure 2), followed by a 5-min wash with DMSO. Then, four cycles of washing, alternating between methanol and DMSO, will be performed.
36. Wash the membrane twice with methanol for 2 min each time. Then, wash the membrane briefly with distilled water for 2 min, twice. Finally, briefly wash the membrane with blocking buffer (5% non-fat dry milk in TBS-T) to prepare it for subsequent steps.
37. Block the membrane with a blocking buffer for 1 h at 25°C.
38. Incubate the membrane with anti-TAMRA mouse antibody diluted 1:1,000 (4 mL per membrane) at 4°C overnight (14–16 h).

**Pause point:** The experiment can be paused here.

### Nascent N-Myc and global protein detection and quantification – Day 10–11

**Timing: 4–6 h**

This section describes the development of a chemiluminescence signal for nascent proteins, allowing for their visualization and quantification. Subsequently, total N-Myc and β-actin are detected to normalize the levels of nascent N-Myc and global proteins, respectively.

39. Wash the membrane with a blocking buffer three times for 5 min each.
40. Incubate membranes with anti-mouse antibodies conjugated with HRP, diluted at 1:10,000 in blocking buffer (4 mL per membrane), for 1 h at 25°C.

***Note:*** We determined that a 1:10,000 dilution of the secondary antibody provides the optimal signal-to-noise ratio. In cases where samples show a low nascent protein signal, the concentration of HRP-conjugated anti-mouse antibody can be increased up to 1:3,000. Conversely, the secondary antibody concentration should be reduced if the signal is excessively strong and risks membrane burn.

41. Wash the membrane with washing buffer three times for 5 min each.

***Note:*** If background noise is observed after signal detection, increase the number of washes to five.

42. Detect signals using enhanced chemiluminescence (ECL) for nascent N-Myc and global proteins. Troubleshooting problems 3, 4, and 5.
  a. Drain the membrane of the washing buffer (ensure it does not dry completely).
  b. Place the membrane in a container and add 0.5–1.0 mL Immobilon Forte Western HRP substrate. Shake horizontally for 90 s.
  c. Carefully tilt the container to drain the excess solution onto a paper towel, not letting the membrane touch the towel to avoid disrupting proteins.
  d. Wrap the blot in plastic wrap or a clean sheet protector to prevent evaporation and light exposure, ensuring no bubbles form. Use a roller if bubbles appear.
  e. Image the blot using the ChemiDoc MP Imaging System with appropriate exposure settings.

**CRITICAL:** Beware of membrane burn due to over-activity of HRP for nascent global protein detection. Troubleshootingproblem 6.

***Note:*** Matrigel used in organoid culture may slightly increase the background signal. Troubleshootingproblem 5.

43. Inactivate HRP^9^ by incubating the membrane in 10% acetic acid for 30 min. Troubleshooting problem 7.

***Alternatives:*** incubate the membrane in 0.1 M glycine-HCl, pH 2.2, supplemented with 1% SDS for 30 min.

44. Briefly wash the membrane with distilled water three times, then incubate it in a blocking buffer for 1 h.

***Note:*** After the washing step, ensure the previously developed signals are completely removed using ECL (repeat step 42). If the signal is still present, repeat step 43 once.

45. To quantify newly synthesized N-Myc protein, probe total N-Myc in immunoprecipitated protein fractions, using total cell lysates as a positive control for N-Myc protein bands. Troubleshooting problem 8.
  a. Incubate the membrane with rabbit anti-N-Myc antibody diluted 1:1,000 in 4 mL of blocking buffer per membrane at 4°C overnight (14–16 h).
  b. Wash the membrane three times with a blocking buffer for 5 min each.
  c. Incubate the membrane with anti-rabbit-HRP antibody diluted 1:3,000 in 4 mL of blocking buffer per membrane for 1 h at 25°C.
46. Wash the membrane three times with washing buffer for 5 min each.

***Note:*** If background noise is observed after signal detection, increase the number of washes to five.

47. Repeat step 42 to detect total immunoprecipitated N-Myc for quantifying nascent N-Myc.
48. To quantify nascent global proteins, probe for actin protein and use it as a loading control.
  a. Incubate the membrane with an HRP-conjugated mouse anti-β-actin antibody diluted 1:20,000 in 4 mL of blocking buffer per membrane for 1 h at 25°C or overnight (14–16 h) at 4°C.
  b. Wash the membrane three times with washing buffer for 5 min each.
    ***Note:*** If background noise is observed after signal detection, increase the number of washes to five.
  c. Detect β-actin using ECL (step 42).
    **CRITICAL:** Beware of membrane burn due to over-activity of HRP for β-actin detection (Troubleshootingproblem 6).

***Optional:*** If studying the effect of treatment on N-Myc expression is required, probe for N-Myc using a rabbit anti-N-Myc antibody following β-actin detection.

***Note:*** Due to distinct molecular weights (β-actin: 45 kDa, N-Myc: 62 kDa), visualization of the N-Myc band is achievable without removing the β-actin signal.

### Data analysis – Day 12

**Timing: 1–4 h**

The following protocol outlines the densitometric analysis of protein bands using Image Lab Software and includes steps for data normalization.

49. Nascent N-Myc proteins: Normalize nascent N-Myc to total N-Myc from immunoprecipitated protein fractions.
  a. Open Image Lab software, click “Open” (Figure 4A), and select the immunoprecipitated total N-myc image file.

**Figure 4:**
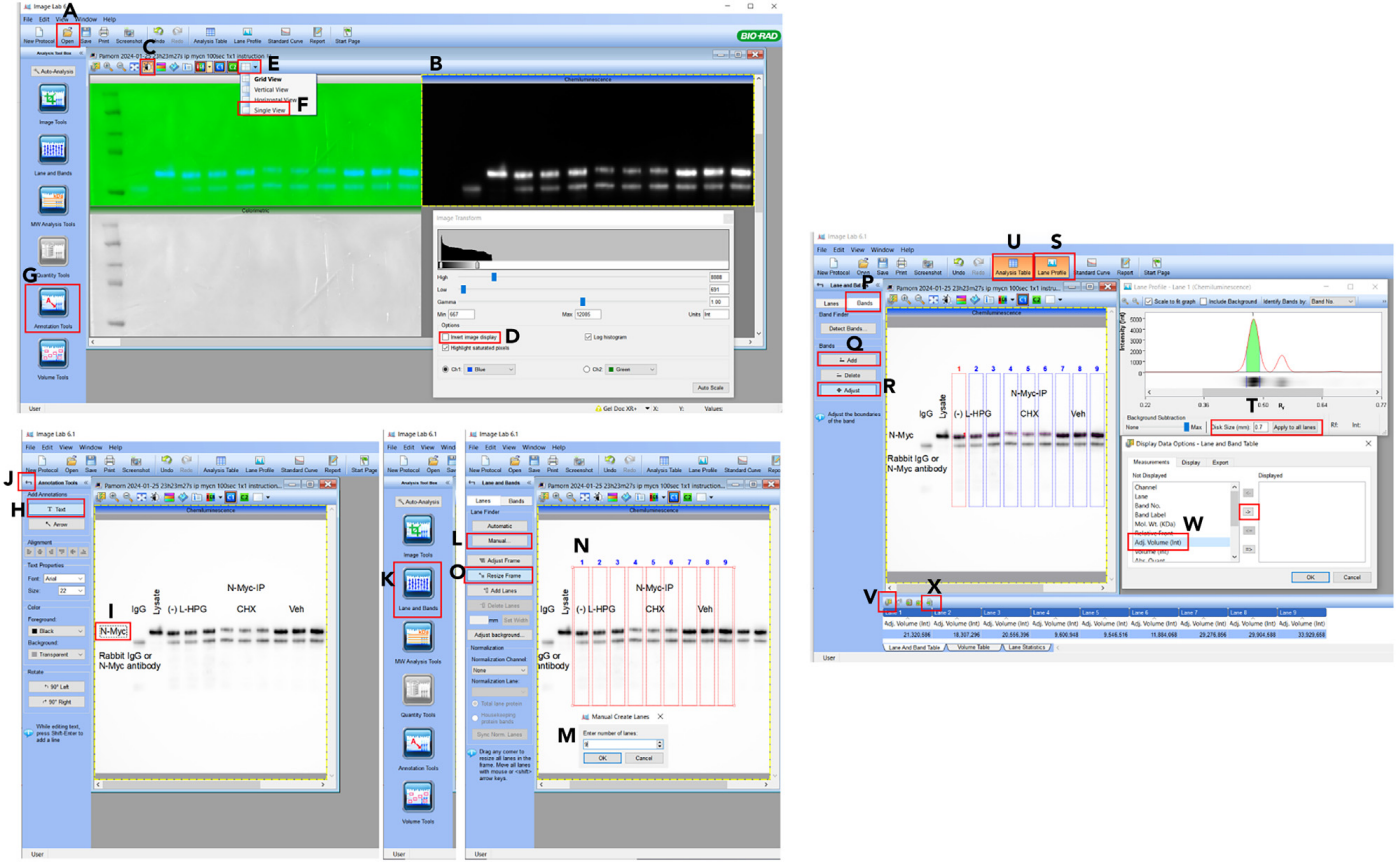
Image Lab Software interface and densitometric analysis workflow of protein bands
  b. Click on the chemiluminescent panel (Figure 4B) and then click on the “Image Transform” panel (Figure 4C). After the pop-up window appears, tick “Invert image display” (Figure 4D) to change the image to a white background.
  c. Adjust the image to a single view (Figures 4E and 4F).
  d. Click “Annotation tools” (Figure 4G) and then “Text” (Figure 4H) to annotate each sample lane and band (Figure 4I).
  e. Exit “Annotation tools” (Figure 4J) and click “Lane and Bands” (Figure 4K).
  f. Click “Manual” to create the lanes (Figure 4L). Enter the number of lanes after the pop-up window appears (Figure 4M). The lanes will appear (Figure 4N). Click “Resize Frame” (Figure 4O) to resize the lanes to fit the protein lanes on the membrane.
  g. Click “Bands” (Figure 4P) and add bands by clicking “Add” (Figure 4Q) and selecting the protein bands. Then, click “Adjust” (Figure 4R) to define the band region (red dotted line). Next, click “Lane profile” (Figure 4S) and adjust the bands to fit the samples. Finally, set background subtraction to 0.7–1.2 mm (Figure 4T).
    ***Note:*** Aim for a clear histogram peak for the protein target band to adjust the background subtraction effectively. In most cases, a value of 0.7 mm works well for single-band analysis.
  h. Click the “Analysis Table” (Figure 4U), then click the display data options icon (Figure 4V) and select “adj. Volume (int)” (Figure 4W). Finally, export the analysis table to Excel (Figure 4X).
  i. Open the image lab file of immunoprecipitated nascent N-Myc. Copy annotations and lanes from the total N-Myc image. For labeling negative controls (samples without L-HPG) with little-to-no signal, create a band with the same width and location as the vehicle lane. Adjust and record the adjusted intensity.
  j. Calculate ratios using the following equation:
    $$Nascent\;N–Myc\;ratio\left(sample\;or\;vehicle\right)=\frac{Nascent\;N–Myc\left(sample\;or\;vehicle\right)}{Total\;N–Myc\;\left(IP\right)}$$
  k. Subtract the background signal from samples and vehicle control using the signal from the labeling negative control (samples without L-HPG).
  l. Normalize across conditions to quantify relative changes in nascent N-Myc protein synthesis using the following equation:
    $$Relative\;nascent\;N–Myc\;\left(sample\;or\;vehicle\right)=\frac{\;Nascent\;N–Myc\;ratio\;\left(sample\;or\;vehicle\right)}{Averages\;of\;nascent\;N–Myc\;ratio\;\left(vehicle\right)\;from\;triplicate\;data\;}$$
    **CRITICAL:** To ensure accurate calculations of average nascent N-Myc ratios for the vehicle control, all triplicate samples must be transferred to the same PVDF membrane. If a vehicle control sample is loaded onto a separate membrane, its corresponding nascent N-Myc protein ratio data can be used for normalization.
  m. Statistical analysis: Use the normalized data from (l) for statistical analysis. Perform a t-test to determine significant differences between experimental groups.
50. Nascent global proteins: Normalize the levels of newly synthesized proteins to the housekeeping protein β-actin. Report the amounts of these nascent proteins from the samples relative to those from the vehicle controls.
  a. Open Image Lab software and open the β-actin image file.
  b. Repeat steps 49a–h (analysis on β-actin image) and record the adjusted signal intensity.
    ***Optional:*** Repeat the analysis from step 49a-h using the N-Myc image file to quantify N-Myc expression.
  c. Open the image file containing nascent global protein data.
  d. Repeat steps 49a–h (analysis on nascent global protein image). Set background subtraction to 90.0 mm (Figure 5).

**Figure 5:**
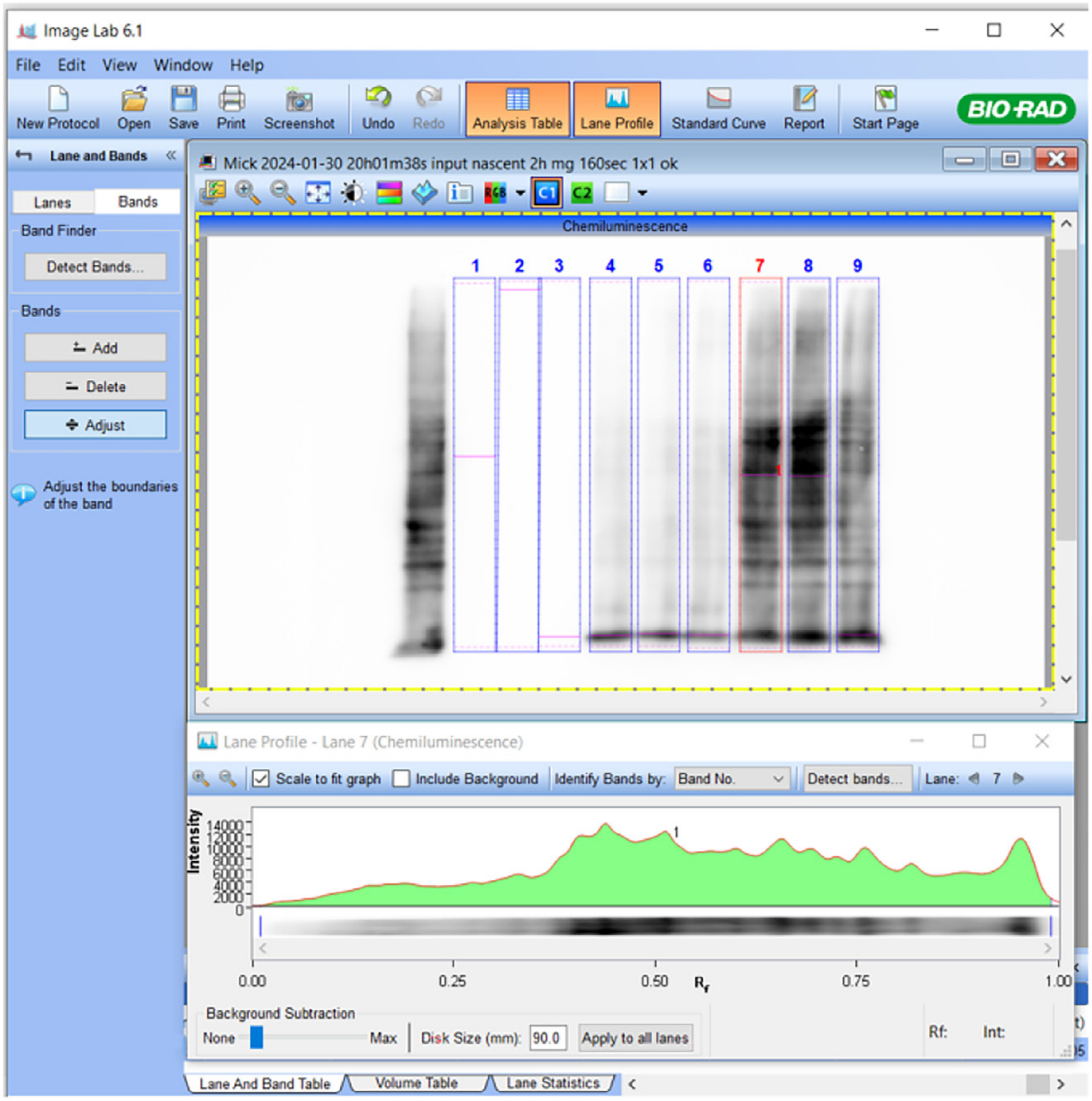
Lane and band adjustment for nascent global protein quantification
  e. Calculate ratios using the following equation:
    $$Nascent\;global\;protein\;\left(or\;N–\text{Myc}\right)\;ratio\;\left(sample\;or\;vehicle\right)=\frac{Nascent\;protein\;\left(or\;N–Myc\right)\;\left(sample\;or\;vehicle\right)}{\beta –actin}$$
  f. Repeat step 49k for nascent global protein quantification.
  g. Normalize across conditions to quantify relative changes in nascent protein synthesis (or N-Myc expression) using the following equation:
    $$Relative\;nascent\;global\;protein\;\left(or\;N–Myc\right)\left(sample\;or\;vehicle\right)=\frac{Nascent\;protein\;\left(or\;N–Myc\right)\;ratio\;\left(sample\;or\;vehicle\right)}{Averages\;of\;nascent\;global\;protein\;\left(vehicle\;\right)\;from\;triplicate\;data}$$
    **CRITICAL:** All triplicate samples must be transferred to the same PVDF membrane for normalization to ensure accurate calculations of average nascent global protein levels for vehicle control. If a vehicle control sample is loaded onto a separate membrane, its corresponding nascent global protein data can be used for normalization.
  h. Repeat step 49m for Statistical analysis using data from (g).

## Expected outcomes

**Figure 6:**
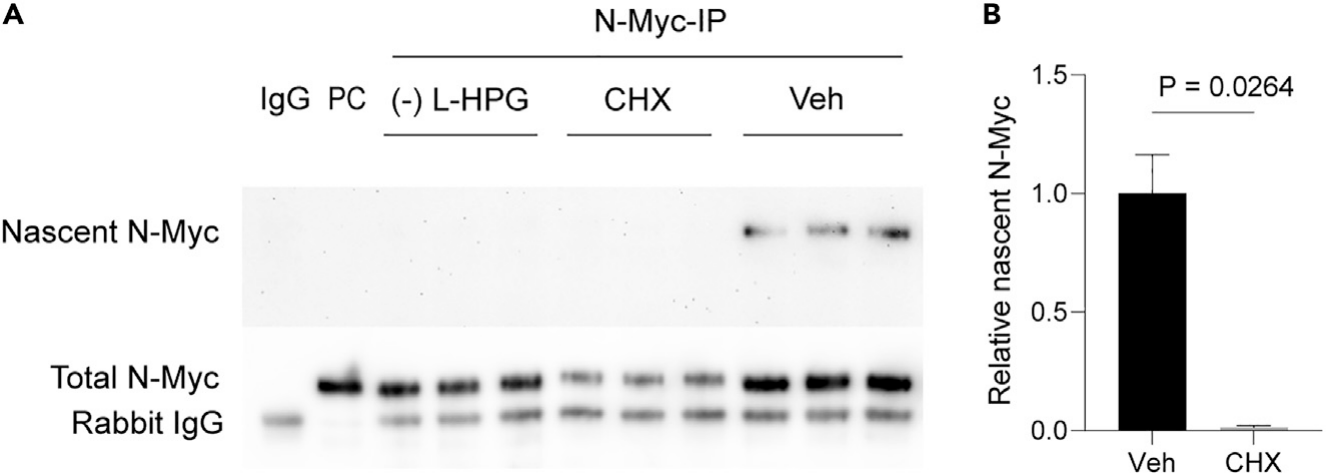
Nascent N-Myc protein detection (A) Detection of nascent and total N-Myc in immunoprecipitated N-Myc proteins (N-Myc-IP) using a Click reaction on PVDF membrane. Following nascent N-Myc detection, the same membrane was used to detect total N-Myc. Abbreviations: CHX: Cycloheximide-treated cells; Veh: Vehicle control (DMSO-treated cells); (−) L-HPG: Cells with non-L-HPG labeling; IgG: Isotype control antibody in immunoprecipitation; PC: Whole protein lysate (positive control for N-Myc band). Protein bands corresponding to rabbit IgG are observed only in the immunoprecipitated proteins where the anti-rabbit secondary antibody (HRP-conjugated) reacts with the immunoprecipitated antibody. (B) Relative nascent N-Myc levels obtained from the data in (A). Welch’s T-test is used to test for statistical significance. Data are presented as mean ± SEM, *n* = 3.

Treatment with CHX, a translation inhibitor, significantly reduced newly synthesized N-Myc (nascent N-Myc) levels in treated cells compared to untreated controls (Figure 6A). Conversely, nascent N-Myc protein bands were absent in immunoprecipitates from samples lacking L-HPG labeling, a metabolic labeling technique for newly synthesized proteins, or using an IgG isotype control antibody (Figure 6A), confirming the specificity of the detection method for nascent N-Myc. Additionally, N-Myc detection from immunoprecipitation is validated by its bands at approximately 62 kDa in the fractions, corresponding to both nascent and total N-Myc. Total N-Myc appeared at the same molecular weight as the positive control band of N-Myc (Figure 6A). The relative levels of nascent N-Myc can be quantified and presented in a bar graph (Figure 6B).

**Figure 7:**
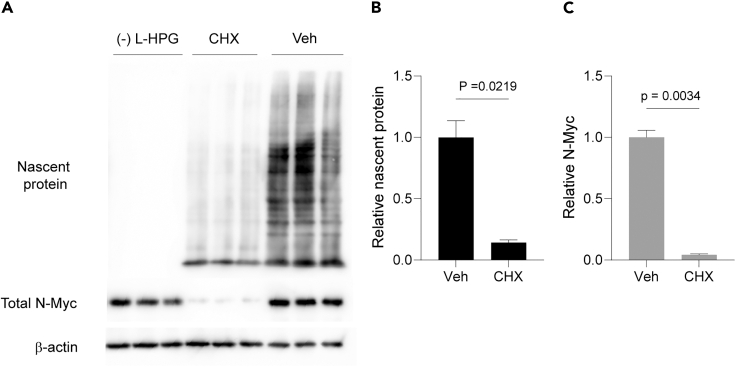
Nascent global protein and N-Myc expression detection (A) Detection of nascent protein (L-HPG-labeled) on PVDF membrane using Click reaction. N-Myc and β-actin were also detected on this membrane following nascent protein detection. Abbreviations: CHX: Cycloheximide-treated cells; Veh: Vehicle control (DMSO-treated cells); (−) L-HPG: Cells with non-L-HPG labeling. (B and C) Relative nascent global proteins and N-Myc expression were obtained from the data in (A). Welch’s T-test and Student’s T-test are used to test statistical significance for quantifying nascent global proteins and N-Myc expression, respectively. Data are presented as mean ± SEM, *n* = 3.

A strong signal in the vehicle control and minimal background in samples without L-HPG labeling indicates successful L-HPG incorporation and nascent global protein detection (Figure 7A). The CHX condition typically shows a reduced signal compared to untreated control (Figure 7A). As expected, N-Myc expression should be present in all conditions, with reduced levels in CHX-treated cells. The relative levels of nascent global proteins (Figure 7B) and N-Myc expression (Figure 7C) can be quantified and presented in bar graphs.

## Limitations

• **L-HPG labeling:** While L-HPG is considered non-toxic, methionine depletion during labeling may induce metabolic changes or stress. Optimizing L-HPG concentration and incubation time is recommended for further labeling experiments.
• **Normalization:** Nascent global proteins are a subset of all proteins. Therefore, total protein normalization offers a more straightforward and representative approach than actin. Total protein normalization can significantly reduce technical variability.^10^ Additionally, total protein is likely less affected in cases where treatments alter actin levels, making it a more reliable control.
• **Applicability:** Our protocol is applicable to other small molecule treatments, cell types, and immunoprecipitated proteins. However, optimization of labeling time and L-HPG concentration is recommended for best results. While on-membrane Click reactions offer enhanced sensitivity through direct detection of labeled proteins, their utility is limited to *in situ* analysis due to challenges in recovering intact proteins for subsequent downstream processes like proteomic studies, unlike in-lysate Click reactions.

## Troubleshooting

### Problem 1

Cells may detach and die after small molecule treatment (related to step 3 in the small molecule treatment), or the cell population used may exhibit a mixture of attached and floating cells.

### Potential solutions

• **Cell death due to treatment:** If cells die due to small molecule or drug treatment, adjust the treatment conditions, such as concentration and duration, to improve cell viability. Dead cells result in low protein concentration, causing significantly lower intensity of β-actin bands in treated samples compared to the vehicle control.
• **L-HPG labeling for mixed cell populations:** For the L-HPG labeling step, in case of a mixture of attached and floating cells, collect the suspension cells by centrifugation at 300–700g for 5 min and pool them with the attached cells before labeling with L-HPG labeling media. Collect the proteins from both forms.

### Problem 2

Protein concentration outside the BCA standard curve range (related to step 12 in determining protein concentration by BCA assay).

### Potential solutions

• **Adjust the dilution factor based on the sample reading:** If the sample reading falls outside the standard curve, adjust the dilution accordingly (either dilute further or use less dilution) to bring it within range. Prioritize higher concentration samples within the curve for better accuracy, especially when multiple dilutions of the original sample are used.

### Problem 3

Low N-Myc signal in immunoprecipitation assay (related to step 42 in the nascent protein detection and quantification).

### Potential solutions

• **Increase input protein lysate:** Consider using more lysate in the IP. Ideally, aim for a concentration of at least 1 μg/μL. This provides more target protein for antibody capture, potentially resulting in a stronger N-Myc band.
• **Optimize antibody concentration:** Try increasing the concentration of the rabbit anti-N-Myc antibody used in the IP. A higher concentration can improve the chances of capturing N-Myc from the lysate. However, it is crucial to perform a titration to determine the optimal concentration that minimizes non-specific binding while maintaining specific N-Myc detection.
• **Utilize antibody validated for N-Myc IP:** Consider using a different antibody specifically validated for N-Myc immunoprecipitation. Antibody performance can vary; some may have a lower affinity or be less suitable for IP than others. Avoid antibodies targeting the methionine-rich region of N-Myc. These regions are less immunogenic and may not be ideal for specific detection.

### Problem 4

Weak nascent protein signal with L-HPG labeling (related to step 42 in the nascent protein detection and quantification).

### Potential solutions

• **Ensure ascorbate solution freshness:** Verify that the solution is freshly prepared immediately before use. Ascorbate loses its effectiveness over time, potentially compromising Click reaction efficiency.
• **Adjust protein loading:** Experiment with loading more protein lysate per well in the Western blot. This increases the starting amount of nascent protein available for detection, potentially resulting in a stronger signal.
• **Optimize L-HPG labeling:** Consider increasing the concentration of L-HPG used for labeling or extending the labeling duration. Experiment with different L-HPG concentrations and incubation times to find the optimal balance for the cell system.
• **Titrate HRP-conjugated antibody:** Try increasing the concentration of the HRP-conjugated anti-mouse antibody (used for nascent protein detection) up to a 1:3,000 dilution.
• **Optimize AGD Concentration:** Consider decreasing the AGD concentration (see Table S1 in the Supplementary file).

### Problem 5

High background in negative L-HPG control (related to step 42 in the nascent protein detection and quantification).

### Potential solutions

• **Reduce secondary antibody concentration:** Consider lowering the concentration of the HRP-conjugated secondary antibody. A lower concentration can decrease non-specific binding events, potentially reducing background signal throughout the blot, including the negative control lane.
• **Optimize cell number in organoid culture (if applicable):** If your experiment utilizes an organoid culture system with Matrigel, try increasing the number of cells seeded per Matrigel drop. This approach aims to increase the overall protein signal in the L-HPG-labeled lanes, improving the signal-to-noise ratio and making the background in the negative control lane less prominent.
• **Adjust ascorbate concentration:** Ascorbate, while necessary for Click reaction, can contribute to background signal if used in excess. Experiment with decreasing the ascorbate concentration to 3 mM (see Table S1 in Supplementary file).
• **Increase washes:** Wash the membranes five times in the washing step before the ECL.

### Problem 6

Membrane burns (dark brown bands) during chemiluminescence (related to steps 42 and 48 in the nascent protein detection and quantification).

### Potential solutions

• **Reduce antibody concentrations:** Consider lowering the concentration of primary antibodies (e.g., anti-β-actin) or HRP-conjugated antibodies (used for nascent protein or β-actin detection).
• **Reduce sample amount:** Experiment with loading less protein lysate per well. This decreases the target protein available for antibody binding, potentially mitigating burns.
• **Alternative chemiluminescent substrate:** Explore using a less sensitive HRP substrate.
• **Optimize incubation time:** Reduce the incubation time with the HRP substrate. Shorter exposure allows for less signal amplification, potentially preventing burns while still capturing a decent signal.

### Problem 7

Persistent signal after HRP inactivation (related to step 43 in the nascent protein detection and quantification).

### Potential solutions

• **Acceptable faint signal:** A faint residual signal, particularly for nascent global proteins, is unlikely to interfere with the downstream analysis. Consider the biological significance of the remaining signal in the context of the experiment.
• **Extended HRP inactivation:** If the remaining signal is problematic, consider extending the incubation time with 10% acetic acid for HRP inactivation (original time: 30 min) to 60 min.

### Problem 8

Overlapping IgG and protein of interest bands (related to step 45 in the nascent protein detection and quantification). The current approach using a secondary antibody (anti-rabbit HRP) introduces an additional band corresponding to the IgG heavy chain, potentially masking the protein of interest bands.

### Potential solutions

• **Use HRP-conjugated primary antibody:** Consider using a primary anti-N-Myc antibody directly conjugated to HRP. This eliminates the need for a secondary antibody, removing the IgG heavy chain band and simplifying the Western blot protocol.

## Resource availability

### Lead contact

Further information and requests for resources and reagents should be directed to and will be fulfilled by the lead contact, Rossukon Kaewkhaw (rossukon.kae@mahidol.edu).

### Technical contact

Technical questions on executing this protocol should be directed to and will be answered by Pamorn Chittavanich (pamorn.chittavanich@outlook.com).

### Materials availability

This study did not generate new, unique materials.

### Data and code availability

This study did not generate new datasets or custom code.

## Acknowledgments

This research project is supported by 10.13039/501100004156Mahidol University (MU’s Strategic Research Fund: 2023).

## Author contributions

P.C. and R.K. contributed to the conceptualization and design. P.C., D.S., A.I.P.S., and R.K. contributed to the methodology. P.C., A.S., and R.K. contributed to data analysis. P.C. and R.K. contributed to the writing – original draft with input from all authors, review, editing, and revising. All authors reviewed the data and approved the final version of the manuscript.

## Declaration of interests

The authors declare no competing interests.
