# Supplementary material for: Protocol for quantifying N-Myc and global protein translation in neuroblastoma cells using click chemistry on polyvinylidene fluoride membranes
Source: STAR Protoc. 2024 Oct 11;5(4):103377. doi: 10.1016/j.xpro.2024.103377 (PMC11735989; doi:10.1016/j.xpro.2024.103377)
Supplement: Document S1. Figures S1–S4 and Table S1 [file mmc1.pdf]

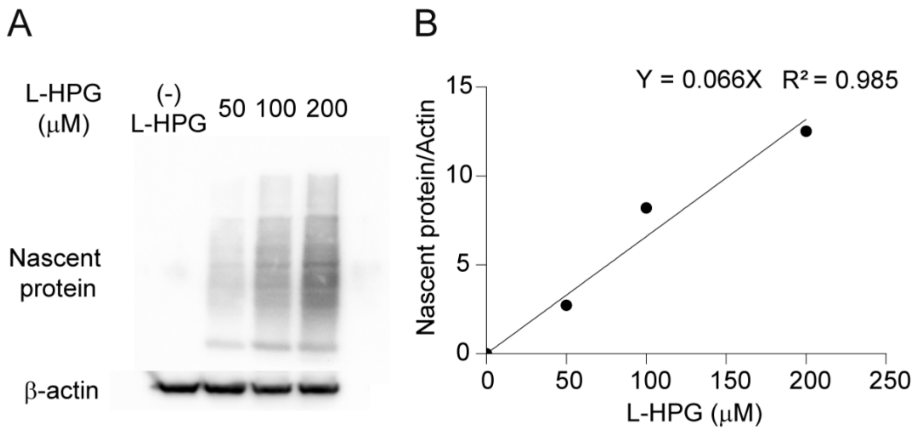

Figure S1: Effect of L-homopropargylglycine (L-HPG) concentration on newly synthesized protein detection, Related to Step 3.

(A) Signal detection of nascent global protein with varying L-HPG concentrations. Cells were labeled with L-HPG for 1 hour. Cells incubated with RPMI-1640 without L-HPG [(-) L-HPG] are negative controls. Actin serves as a loading control.

(B) Simple linear regression for the ratio of the signal intensity of nascent global protein translation to those of actin in (A), with a slope of 0.066.

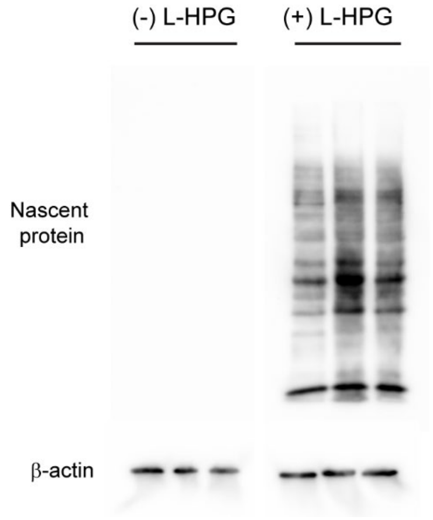

Figure S2: Signal-to-noise ratio achieved with 30-minute L-homopropargylglycine (L-HPG) labeling for nascent protein detection in neuroblastoma cells, Related to Step 3.

A sufficient signal-to-noise ratio for nascent global protein detection is consistently achieved after 30 minutes of labeling with 200 µM L-HPG [(+) L-HPG], the minimal time tested in this protocol. Cells incubated with RPMI-1640 without L-HPG [(-) L-HPG] serve as a background control. Actin serves as a loading control. Lanes were not continuous (a gap between (-) L-HPG and (+) L-HPG) but were run on the same gel and blotted on the same membrane.

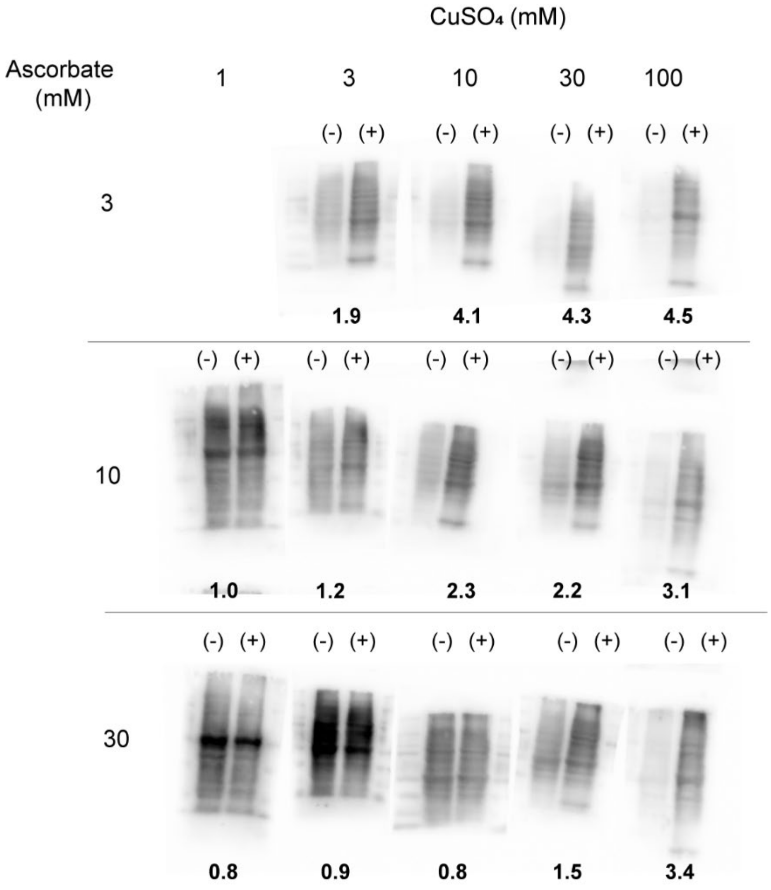

Figure S3: Optimization of CuSO4 and ascorbate concentration for click reaction, Related to Step 33.

This figure shows the optimization of the CuSO4 and ascorbate concentrations for the click reaction following the L-HPG labeling of nascent proteins in neuroblastoma cells. Cells were labeled with (+) or without (-) L-HPG, and signal intensity was detected using a click reaction with various concentrations of CuSO4 and ascorbate. The numeric value displays the signal-to-noise ratio, calculated as the ratio of the intensity from L-HPG-labeled cells (+) to that from non-labeled cells (-). The reaction containing three mM ascorbate and a range of 10-100 mM CuSO4 resulted in high signal-to-noise ratios (4.1-4.5). The choice of three mM ascorbate and 20 mM CuSO4 was based on the balance between achieving a high signal-to-noise ratio and minimizing potential side reactions. These concentrations were chosen for further optimization.

A

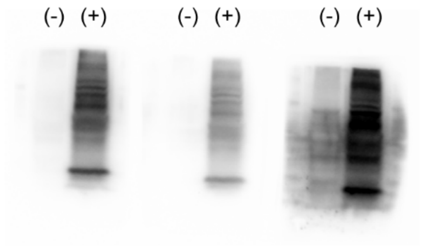

$CuSO_4$ (mM)	20	20	20
Ascorbate (mM)	3	3	3
AGD (mM)	-	10	-
THPTA (mM)	-	-	10
(+) L-HPG Intensity	12.9	6.6	20.6
(-) L-HPG Intensity	1.0	0.4	10.0
(+) L-HPG/(-) L-HPG Signal-to-noise ratio	12.9	16.5	2.1

B

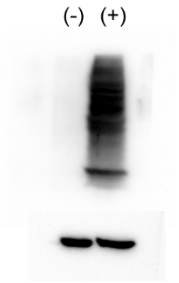

$CuSO_4$ (mM)	20
Ascorbate (mM)	10
AGD (mM)	10
(+) L-HPG Intensity	16.0
(-) L-HPG Intensity	1.1
(+) L-HPG/(-) L-HPG Signal-to-noise ratio	14.5
(+) L-HPG/(-) L-HPG Signal-to-noise ratio (with actin normalization)	13.3

Figure S4: Effect of aminoguanidine (AGD) and tris(hydroxypropyl)triazolylmethylamine (THPTA) on click reaction signal-to-noise ratio, Related to Step 33.

(A) Impact of AGD and THPTA on click reaction: This panel shows how AGD and THPTA influence the click reaction for nascent protein detection. AGD acts as an inhibitor by forming a complex with copper ions (slows the generation of catalytically reactive Cu^{+} ions from Cu^{2+}), potentially reducing non-specific background signals. THPTA, on the other hand, binds to copper ions to maintain their active state (similar to ascorbate) and reduce cellular toxicity. Both additives were included in the reaction to optimize the signal-to-noise ratio, calculated as the ratio of intensity from L-HPG-labeled cells (+) to that from non-

labeled cells (-). The results show that AGD decreased the overall signal intensity for both labeled (+) and non-labeled (-) cells but led to an improved signal-to-noise ratio by reducing background noise. Conversely, although THPTA (10 mM) increased the intensity of the nascent protein signal, it also significantly reduced the signal-to-noise ratio by amplifying the background noise.

(B) Click reaction with AGD for optimized signal-to-noise ratio: Based on panel (A) findings, AGD was chosen for further optimization due to its potential for providing a lower background signal. In this optimized formulation, the ascorbate concentration was increased to enhance the overall signal, resulting in an apparent signal-to-noise ratio. Actin band intensity serves as a loading control.

Table S1: Click reaction and detection agents for nascent protein, Related to Step 33.

**Reagent**	**Function**	**Concentration**
L-Homopropargylglycine (L-HPG)	Serve as metabolic labeling agent. It is incorporated into nascent proteins during cell culture, introducing an alkyne group for subsequent CuAAC (copper-catalyzed azide-alkyne cycloaddition) click chemistry.	Optimum concentration: 200 µM
$CuSO_4$	The catalyst for the CuAAC click reaction	Optimum concentration: ≥ 10 mM
TAMRA-azide	It is a bioconjugation reagent that utilizes CuAAC click chemistry to label L-HPG incorporated in nascent protein translation.	Fix to 10 µM
DMSO	Help TAMRA-azide solvate throughout a PVDF membrane	Without DMSO, TAMRA-azide exhibits non-specific absorption onto the PVDF membrane
AGD	Act as an inhibitor by forming a complex with copper ions, potentially reducing non-specific background signal	• Optimum concentration: 5-10 mM in the reaction with 20 mM $CuSO_4$, 10 mM ascorbate<br>• 20 mM concentration can suppress the click the reaction
Ascorbate (vitamin C)	A reducing agent in the CuAAC click reaction maintains Cu+ in its active state (reducing Cu^{2+} to Cu^{1+} to initiate the reaction)	Optimum concentration: 3-10 mM (not exceeding the $CuSO_4$ concentration)
Primary antibody (anti-TAMRA)	Recognizes TAMRA proteins (detects L-HPG-labeled nascent proteins).	Fix to 1:1,000
Secondary antibody (HRP-conjugated antibody)	An antibody that binds to the primary antibody and is conjugated to HRP for chemiluminescent detection	Optimum concentration: 1:10,000 dilution for our optimized reaction
